# Supporting Seamless Mobility for P2P Live Streaming

**DOI:** 10.1155/2014/134391

**Published:** 2014-05-19

**Authors:** Eunsam Kim, Sangjin Kim, Choonhwa Lee

**Affiliations:** ^1^Department of Computer Engineering, Hongik University, Seoul 121-791, Republic of Korea; ^2^Hyundai Autoever Corporation, 576 Sam, Uiwang, Gyeonggi 437-040, Republic of Korea; ^3^Division of Computer Science and Engineering, Hanyang University, Seoul 133-791, Republic of Korea

## Abstract

With advent of various mobile devices with powerful networking and computing capabilities, the users' demand to enjoy live video streaming services such as IPTV with mobile devices has been increasing rapidly. However, it is challenging to get over the degradation of service quality due to data loss caused by the handover. Although many handover schemes were proposed at protocol layers below the application layer, they inherently suffer from data loss while the network is being disconnected during the handover. We therefore propose an efficient application-layer handover scheme to support seamless mobility for P2P live streaming. By simulation experiments, we show that the P2P live streaming system with our proposed handover scheme can improve the playback continuity significantly compared to that without our scheme.

## 1. Introduction

With the widespread deployment of high speed broadband networks such as FTTH, the IPTV services converging broadcasting and communication technologies have emerged. So far most commercial IPTV systems have employed the client/server architecture where video data are transmitted only from servers to clients. To support a huge number of IPTV subscribers at the same time, however, the client/server architecture should employ CDN (content distribution networks) structures to reduce data transmission delay. As the number of IPTV subscribers increases, the client/server architecture thus causes high expense for expanding network capacity by adding proxy servers to accommodate all the increasing subscribers [1]. The personalized IPTV services including time-shifted TV may make it even more difficult for IPTV systems to manage network traffic efficiently [2, 3].

Many research efforts have therefore been made on peer-to-peer (P2P) live streaming since it is a cost-effective and scalable alternative to client/server architectures on the Internet [4–6]. In P2P live streaming systems, peers exchange distributed video data with each other on virtual overlay networks. Thus, the better performance can be achieved as the number of participating peers increases.

On the other hand, with recent advance in wireless networks and advent of powerful mobile devices such as smart phones, the video streaming services have become feasible in mobile platforms [7–9]. Since these mobile IPTV services can provide users with the mobility and portability in wireless networks, the users' demand to enjoy IPTV services with mobile devices has been increasing rapidly. One of the most challenging issues when designing mobile IPTV systems is that users may experience the degradation of service quality due to data loss caused by the handover occurring when mobile devices are moving across APs. To get over this problem, it is thus essential to provide seamless mobility for P2P live streaming.

To minimize the transmission delay during the handover period, many schemes have been proposed at different layers of the protocol stack including data link [10, 11], network [12, 13], and transport layer [14, 15] depending on the characteristics of each layer. In the handover schemes at the layers below the application layer, however, the data loss inherently occurs while the network is being disconnected to switch APs. To avoid playback jitter in P2P live streaming systems, it is thus important to compensate for the amount of data that could not be received during the handover period. We thus need a new handover scheme at the application layer apart from that at lower layers. In fact, several application-layer handover schemes have been proposed recently but they did not consider P2P streaming structures, all based on the client/server architecture using CDN structures with low scalability and high cost.

In this paper, we therefore propose an efficient application-layer handover scheme to provide seamless mobility in the presence of handover by considering mobile peers' limited resources and the unstable characteristics of wireless networks. In our proposed scheme, to receive data from neighbor peers at a faster speed, neighbor peers transmit data to a mobile peer through a push manner for the period around the handover. To further improve the performance, an agent peer for the mobile peer is selected among stationary peers. The agent peer receives data in place of the mobile peer before and during the handover and then transmits the data through a new AP after the handover. It can transmit data at a faster speed compared to the other neighbor peers because it is selected depending on its RTT value from a new AP and the appropriateness of its buffered period from a mobile peer's perspective.

Through extensive simulations, we demonstrate the effectiveness of our proposed handover scheme. The simulation results show that the mobile P2P streaming system with our handover scheme improved the playback continuity significantly compared to that without our scheme. We also show that we can further improve the performance by adjusting the weight value used when selecting an agent peer depending on peers' current situation.

The remainder of this paper is organized as follows.  Section 2 describes related work to mobile IPTV systems and handover schemes.  Section 3 describes our design considerations to develop an efficient handover scheme.  Section 4 proposes an efficient handover scheme for mobile P2P live streaming systems.  Section 5 presents extensive simulation results. Finally, Section 6 offers conclusions.

## 2. Related Work

So far most streaming systems have employed the client/server architecture based on content delivery networks (CDNs) that consist of many proxy servers geographically distributed on the Internet. However, this architecture requires tremendous cost to expand the network capacity for the rapidly increasing number of IPTV subscribers. To solve this scalability problem, many P2P streaming systems have thus been proposed. They can be broadly classified into tree-push and mesh-pull structures. The tree-push structures require much overhead to rebuild tree structures whenever peers join or leave [4]. On the other hand, the mesh-pull structures provide robust structure against peers' churn while creating long startup delay and requiring a large number of data exchanges among peers [5]. Thus, several hybrid push-pull architectures such as mTreebone [6] have been proposed to offer a good tradeoff between two structures. However, these P2P structures did not consider mobile platforms in a wireless network environment, only having focused on constructing overlay networks using stationary peers in a wired network environment.

With recent bandwidth improvement in wireless networks and advent of various mobile devices, mobile IPTV systems have become feasible. So far most of research efforts on mobile P2P streaming systems have been made in MANET [16] or iMANET [17]. However, they are not suitable for large-scale P2P systems due to their characteristics of high energy consumption and low scalability caused by direct communication between peers. To provide IPTV services with mobile devices, some systems have attempted to simply add mobile devices to the existing P2P streaming structure based on wired networks via APs [18]. As a result, they did not consider the characteristics of mobile computing environment such as low bandwidth, unstable wireless signal, and peers' high mobility when designing the mobile P2P live streaming systems.

On the other hand, many handover schemes have been proposed at each protocol layer to support mobile peers' mobility. The handover schemes at the data link layer have focused on performing the fast handover between two APs according to their signal strength using RSSI (received signal strength indication) [10, 11]. At the network layer, the proposed schemes have attempted to reduce the handover period by receiving CoA (care of address) as quickly as possible [12, 13]. To do so, they predict mobile peers' moving directions when they move from one subnet to another subnet. At the transport layer, new protocols such as mSCTP and mDCCP to add mobility features to the existing TCP and UDP protocols have been proposed [14, 15]. In these handover schemes operating at the layers below the application layer, however, it is not possible to avoid data loss because the network is physically disconnected during the handover period. To compensate for such data loss at lower layers, several application layer handover schemes have therefore been developed apart from lower layer schemes [19–21]. However, those application layer handover schemes have been developed based only on CDN structures, not considering the P2P live streaming structures.

## 3. Design Considerations for Seamless Mobility

We describe several considerations to reflect the characteristics of mobile devices and wireless networks when designing an efficient application-layer handover scheme for P2P live streaming systems.

### 3.1. Data Transmission Manners for P2P Live Streaming

In a mesh-based P2P streaming architecture, the data unit for data delivery and display is a video block. Each video is divided into small blocks, which are distributed to other peers through the mesh structure. Each peer displays video blocks after buffering and sequencing received blocks in memory. Peers periodically exchange their status using buffer maps that represent the blocks' availability in peers' buffers. After obtaining buffer maps from its neighbors, a peer can determine to which neighbor peers it will request missing blocks. In such a mesh-based streaming structure, where data are transmitted in a pull manner, playback should be delayed until a peer can receive the sufficient data to start to playback a video.

On the other hand, in a tree-based P2P streaming architecture, peers receive video data from an origin server or parent peers only in a push manner. This structure enables peers to transmit data at a faster speed because they can keep transmitting data without any specific requests from their child peers once the tree structure is constructed.

In general, a mobile peer tends to experience data loss while communicating with others due to unstable wireless network environment. The mesh structure is thus more suitable for mobile P2P streaming architecture since a mobile peer can receive data more stably by requesting the retransmission of lost blocks. However, a peer has the longer delay when receiving data in a pull manner compared to that in a push manner. This is because, in a pull manner, it can receive the desired blocks from neighbor peers by specifically requesting them after exchanging buffer maps. Moreover, since a mobile peer cannot receive any data block during the handover, the transmission delay can be much longer after the handover. In our P2P live streaming system, a mobile peer therefore receives data in a push manner only for a short period around the handover to receive data at a faster speed.

### 3.2. Criteria for Selecting Neighbor Peers for a Mobile Peer

Even though a mobile peer is not able to receive any data only for a short period due to network condition, especially during the handover, it must continue to playback the video, keeping consuming the data that have been buffered before that period. As a result, a mobile peer may experience playback jitter unless it can quickly obtain the required data as soon as the network is available. To avoid such degradation of playback quality in our P2P live streaming system, we consider the proximity to a mobile peer as the first criterion when selecting neighbor peers. This can reduce the network latency through the shortened transmission route.

On the other hand, peers buffer the data corresponding to a specific period around their current playback positions. Since lag times between an origin server and peers are getting large as the number of peers increases, however, peers' buffering periods also become widely distributed. Furthermore, when supporting VCR operations, peers' playback positions become distributed more widely. Note that a mobile peer can receive data at a faster speed as neighbor peers are buffering more data required for its immediate playback. The other criterion is therefore how much data required by a mobile peer a candidate peer is currently buffering.

### 3.3. Handover Prediction

If a mobile peer cannot receive sufficient data before the handover, they may not be able to continue playing back the video due to lack of buffered data even though they receive the data at a fast speed after the handover. To prevent this situation, it is necessary to receive as much data as possible by predicting the handover before it actually happens. In our P2P live streaming system, we adopt the most common technique using signal strength of APs, that is, RSSI, to predict the handover. In other words, a mobile peer predicts that handover will occur soon when the difference of signal strength between the current and the target AP becomes smaller than the given threshold. Once the handover is predicted, neighbor peers can transmit data to the mobile peer at a faster speed by switching their transmission manner to a push one.

## 4. An Efficient Handover Scheme for P2P Live Streaming

In this Section, we propose a new application-layer handover scheme to provide the seamless mobility in P2P live streaming systems. We first describe our handover behavior model according to the state of each peer. We then explain our agent peer selection policy to minimize playback jitter.

### 4.1. Handover Behavior Model

#### 4.1.1. State Transition for Handover Behavior

In our proposed handover scheme, there are four states for each mobile peer: normal (*N*), prediction (*P*), handover (*H*), and after-handover (*A*) state. When a mobile peer is triggered by one of several events, it changes its state after taking the corresponding action depending on its current state as follows.

- The *N* state represents the state where the signal of the current AP measured by a mobile peer is stronger than those of other adjacent APs by the threshold value for handover prediction.
- The *P* state indicates the period between after the handover is predicted to occur and before the handover actually occurs.
- The *H* state indicates the period when a mobile peer is switching its current AP to a new one while the network is being disconnected due to the handover.
- The *A* state represents the period when a mobile peer is receiving data through a newly connected AP for a short period until going to *N* state.

**Figure 1:**
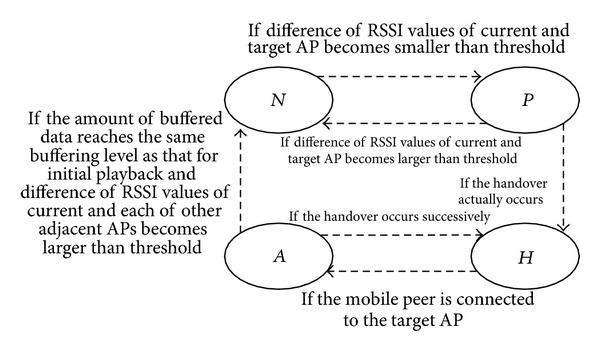
State transition diagram for handover behavior.

Figure 1 shows a state transition diagram of a mobile peer for its handover behavior. A mobile peer can move to another state depending on the events relating to the handover. First, a mobile peer usually enters into *N* state when it joins. When the difference of RSSI values of the current and target AP from the mobile peer becomes smaller than the threshold, it transits to *P* state. When the handover actually occurs, its state moves to *H* state. If the signal of the target AP from the mobile is getting weaker at *P* state, it returns to *N* state. Once the mobile peer is connected to the target AP, it transits to *A* state. Its state is changed to *N* state when the following two requirements are met: one is that the amount of buffered data should reach the same buffering level as that for initial playback and the other is that the difference of RSSI values of the current and each of other adjacent APs should become larger than the threshold value. If the handover occurs successively at *A* state, it returns to *H* state.

#### 4.1.2. Data Transmission Route and Manner

**Figure 2:**
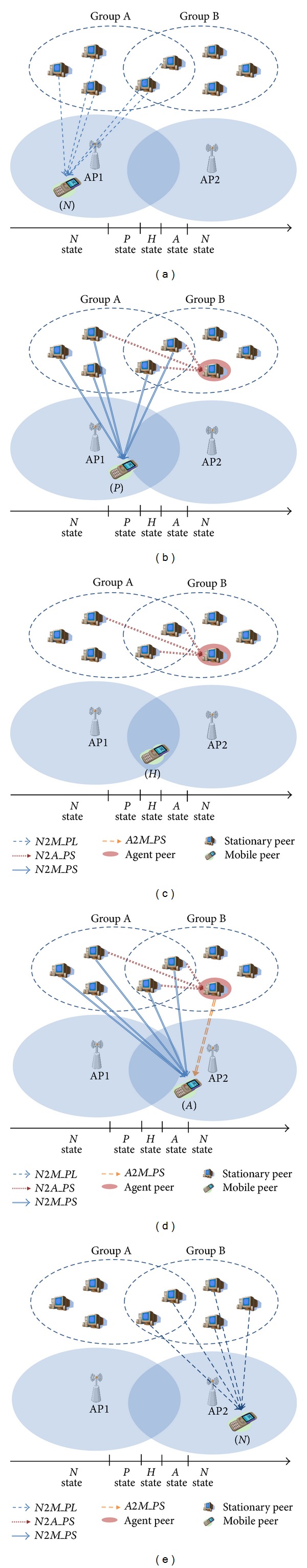
Data transmission route and manner according to a mobile peer's state: (a) *N* state before handover, (b) *P* state, (c) *H* state, (d) *A* state, and (e) *N* state after handover.

In our P2P streaming system, a mobile peer has different data transmission route and manner according to its current state. As shown in Figure 2(a), at *N* state, it receives data from neighbor peers in a pull manner (*N2M_PL*: neighbors to mobile in a pull manner). To maximize the amount of data that can be received at *P* state before the handover, the mobile peer receives data from neighbor peers after switching the transmission manner to a push one (*N2M_PS*: neighbors to mobile in a push manner) as shown in Figure 2(b). To transmit data to the mobile peer at a faster speed after the handover, an agent peer also receives as much data as possible at this state. The agent peer thus receives data from neighbor peers in a push manner (*N2A_PS*: neighbors to agent in a push manner).

As shown in Figure 2(c), at *H* state, the mobile peer cannot receive any data during the handover while the agent peer maintains* N2A_PS* because it can still receive data from neighbor peers. The mobile peer transits to *A* state once it is connected to a new AP. It receives data as fast as possible from the agent peer as well as from neighbor peers in a push manner to minimize playback jitter (*N2M_PS*,* A2M_PS*: agent to mobile in a push manner) as shown in Figure 2(d). After returning to *N* state, the mobile peer receives data from newly selected neighbor peers in a pull manner (*N2M_PL*) as shown in Figure 2(e).

### 4.2. Agent Peer Selection Policy

To further improve the playback continuity of a mobile peer when the handover occurs, a tracker server selects an agent peer for the mobile peer among stationary peers. In our P2P live streaming system, the agent peer plays an important role in reducing playback jitter caused by the handover. The agent peer receives data in place of the mobile peer from the moment the handover is predicted until the handover ends. It then transmits the buffered data to the mobile peer as fast as possible through a new AP so that the mobile peer cannot experience buffer starvation. To select the most suitable peer as an agent peer to perform this task, we thus consider a couple of criteria: RTT value and the appropriateness of the buffered period. The RTT value is considered to measure the transmission delay between a new AP and a candidate peer. The appropriateness of buffered period is considered to estimate how appropriate the period of the data buffered in a candidate peer is for the immediate playback of a mobile peer. In other words, it indicates how much required data from a mobile peer's perspective a candidate peer is currently buffering. The following equation represents the criteria to select an agent peer for a mobile peer:

(1) $$\begin{array}{c} \text{MIN}\left\{W\times {\text{RTT}}_{i}+\left(1-W\right)\times {\text{ABP}}_{i}\right\}. \end{array}$$

In (1), *i* is an index for a specific candidate peer, RTT_*i*_ and ABP_*i*_ denotes the RTT value from a new AP and the appropriateness of the buffered period of a candidate peer with an index of *i*, respectively, and *W* is the weight value between RTT_*i*_ and ABP_*i*_. The candidate peer with a minimum value of (1) is selected as an agent peer for the corresponding mobile peer.

It is noted that *W* can be adjusted according to peers' current situation. If the network latency affects the performance more significantly in some situation, we need to increase *W*. On the contrary, in the situation where the appropriateness degree of buffered data for the playback of the mobile peer is a more important factor to improve the performance, it is necessary to decrease *W*.

## 5. Experimental Evaluation

To show the effectiveness of our proposed handover scheme for mobile P2P live streaming systems, we have performed extensive simulations using a QualNet network simulator. The default values of simulation parameters are shown in Table 1. They are used throughout our simulations unless otherwise indicated. It is assumed that mobile peers are moving at a speed of the range from 5 to 20 Km/h and the latency range of the handover is from 0.5 to 1 second. The bandwidths of backbone, wired, and wireless networks are set to 100 Gbps, 100 Mbps, and 20 Mbps, respectively. Each video has 750 Kbps playback rate. The numbers of peers are 1000 and each peer can have at most 5 neighbor peers. The RTT values between peers range from 1 to 200 ms and the average buffering interval starting from the current playback positions of peers is 3 seconds. The weight value of (1), that is, *W*, is set to 0.5.

### 5.1. Effectiveness of Our Handover Scheme

**Figure 3:**
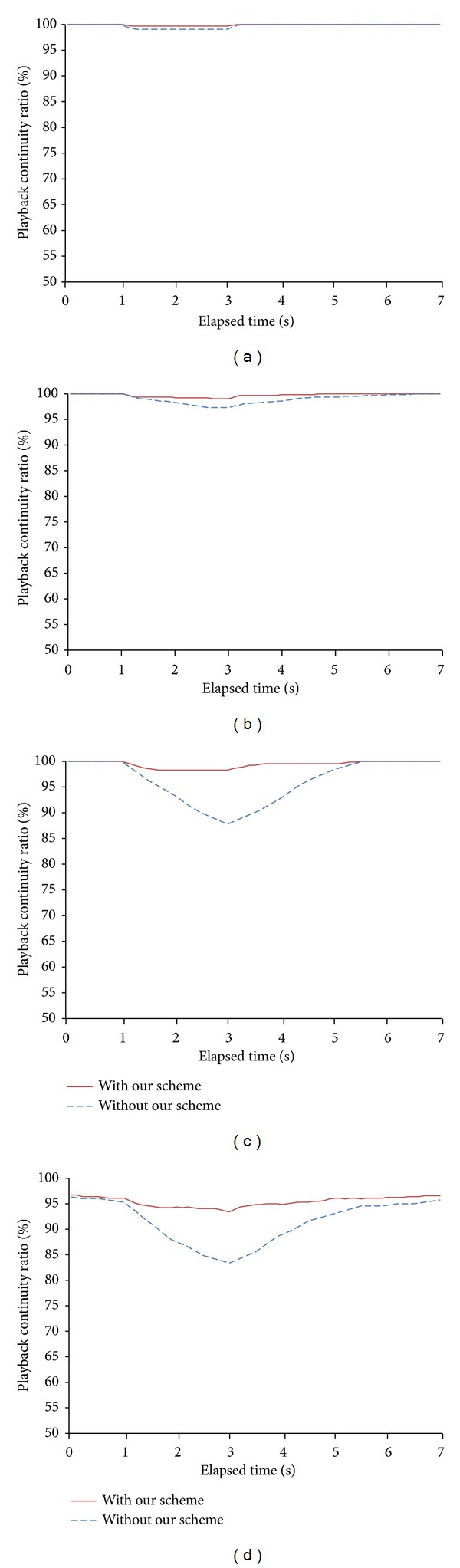
Playback continuity ratios of two cases according to effective wireless network bandwidth: (a) with effective bandwidth of 16 Mbps, (b) with effective bandwidth of 14 Mbps, (c) with effective bandwidth of 12 Mbps, and (d) with effective bandwidth of 10 Mbps.

Figure 3 shows the comparison of playback continuity ratios for 7 seconds around the handover in two cases: with and without our proposed scheme. The case without our proposed scheme indicates the existing P2P live streaming systems based on mesh structure that do not perform any particular operation for the handover. To examine the impact of effective wireless network bandwidths on the performance, we varied them from 16 Mbps to 10 Mbps by generating background traffic from 4 to 10 Mbps. You can see that the handover occurs immediately after one second position from the beginning of the *x*-axis in Figure 3.

The experimental results show that the case with our handover scheme improved the playback continuity ratios significantly compared to the case without our scheme. Especially, in case of effective bandwidth of 10 Mbps in Figure 3(d), the difference of the minimum playback continuity ratios between two cases was 10.1%. That is, the minimum playback continuity ratio of the case with our scheme was 93.4% while that of the case without our scheme was only 83.3%. This implies that our handover scheme performs effectively no matter how much bandwidth the wireless network provides. This is possible because our handover scheme can obtain the sufficient amount of data required by a mobile peer in advance through handover prediction and an agent peer. The mobile peer can also rush to receive data after the handover by adopting a push transmission manner.

It can also be seen from Figure 3 that, as the effective bandwidth of wireless networks decreases, that is, as the background traffic becomes heavier, the playback continuity ratios of two cases also decrease. It is noted that, however, the performance degradation degree in the case with our handover scheme is much lower than that without our scheme. As the background traffic increases from 4 Mbps to 10 Mbps, the minimum playback continuity ratio of the case with our scheme was reduced by only 6.3% while that without our scheme was reduced by 15.7%. This result indicates that our handover scheme can utilize the decreased network bandwidth efficiently. That is, a mobile peer can overcome the shortage of the network bandwidth after the handover by receiving as much data as possible before and during the handover together with an agent peer. It can also reduce the transmission latency and the number of control messages considerably by receiving data in a push manner for the period around the handover.

### 5.2. Impact of Agent Peer Selection Criteria

To investigate the impact of two criteria in (1) on the performance when selecting an agent peer, we have made three different distributions for each criterion. The RTT values between a mobile peer and other candidate peers are distributed as follows: narrow distribution (5–100 ms), average distribution (1–200 ms), and wide distribution (0.1–1000 ms). The lengths of peers' average buffering intervals starting from the current playback positions are also distributed as follows: short distribution (1 second), average distribution (3 seconds), and long distribution (5 seconds). In our simulations, peers' RTT values and buffering interval lengths are randomly generated within the given range of each distribution. We also set the effective wireless network bandwidth to 12 Mbps for these simulations.

**Figure 4:**
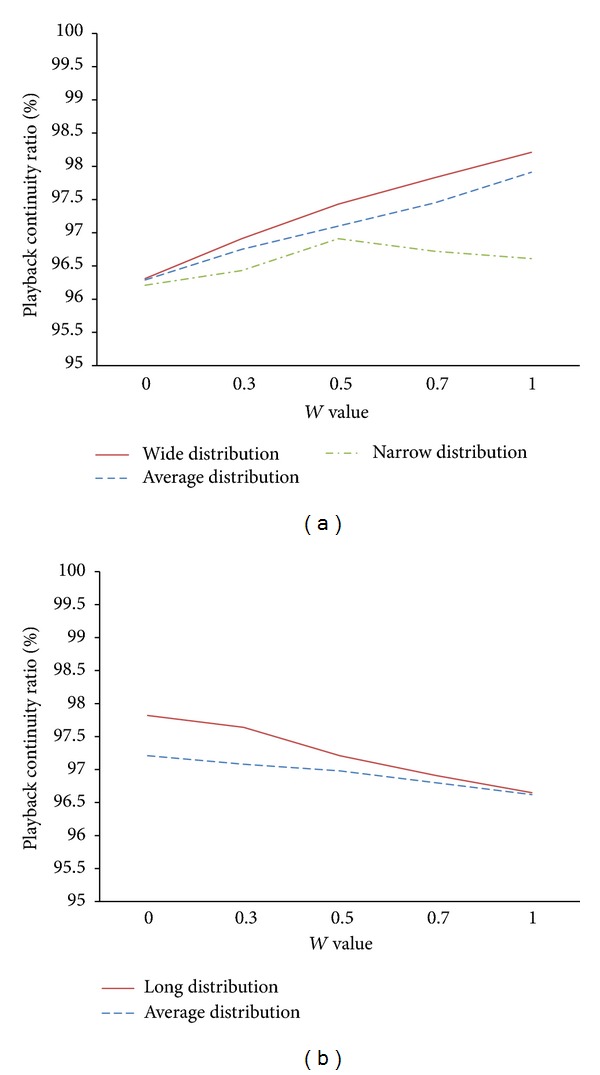
Impact of two criteria for agent peer selection on the playback continuity ratio: (a) RTT values and (b) lengths of average buffering interval.

Figure 4(a) shows the playback continuity ratios according to the distribution degree of RTT values while fixing the distribution degree of buffering interval lengths to a short one. In case we employ wide and average distribution for buffering interval lengths, we achieved the highest playback continuity ratio when *W* is 1 while achieving the lowest one when *W* is 0. The differences between the highest and lowest ratio in case of wide and average distribution are 1.9% and 1.6%, respectively. Note that, as *W* increases, the playback continuity ratios also keep increasing almost linearly. This implies that, as peers are distributed more widely, that is, as the differences in RTT values are getting larger, it is more advantageous to select the peer with shorter RTT value from the mobile peer as an agent peer as indicated in (1). When using the narrow distribution for buffering interval lengths, the playback continuity ratio was highest when *W* is 0.5 while it was lowest when *W* is at both ends 0 and 1. This indicates that the short distribution of RTT values and the narrow distribution of buffered interval lengths have similar degree from the perspective of the agent peer selection criteria. In such peers' situation, we thus need to consider two criteria to the same degree to maximize the performance.

Figure 4(b) shows the performance according to the distribution degree of buffering interval lengths when the distribution degree of RTT values is fixed to a narrow one. The simulation results show similar trends to those in Figure 4(a) except that the performance improved with the decreased value of *W*. That is, in case of the long and average distribution for RTT values, we achieved the highest playback continuity ratio when *W* is 0 while achieving the lowest one when *W* is 1. The improvement ratios in the long and average distribution are 1.2% and 0.6%, respectively. It can also be seen that the performance keeps improving with the decreased value of *W*. This indicates that, as average buffering interval lengths of peers are getting longer, it is more beneficial to select the peer that are buffering more data required by the mobile peer. In this case, we thus need to put more weight on the appropriateness of buffering period as indicated in (1).

From the simulation results in Figure 4, we have observed the impact of two criteria including RTT values and the appropriateness of the buffered periods when selecting an agent peer. Note that we can make the agent peer selection policy flexible in different peers' situation by adjusting *W* value, thereby further improving the performance.

## 6. Conclusions

We have presented an efficient application-layer handover scheme in mobile P2P live streaming systems to improve the playback continuity ratio significantly even though the handover occurs. This improvement was possible because a mobile peer can receive the sufficient amount of data required for the video playback in advance through handover prediction and an agent peer. It can also receive data at a faster speed for the period around the handover by employing a push transmission manner. As the video contents requiring the higher playback rate, such as 3D and UD (ultrahigh definition) TV, are emerging, our handover scheme is expected to be widely applied to many applications in mobile platforms to provide seamless mobility even though the handover occurs.

## Figures and Tables

**Table 1:** Summary of simulation parameter values.

Parameter	Default value
Moving speed of mobile peers	5~20 Km/h
Handover latency	0.5~1 second
Bandwidth of backbone networks	100 Gbps
Bandwidth of wired networks	100 Mbps
Bandwidth of wireless networks	20 Mbps
Video playback rate	750 Kbps
Number of peers	1000
Number of neighbor peers	5
RTT values	1–200 ms
Buffering interval	3 seconds
*W*	0.5
